# NeuroVision training may improve subjective visual quality in patients with residual refractive error after SMILE: a prospective clinical trial

**DOI:** 10.3389/fnins.2026.1852368

**Published:** 2026-09-09

**Authors:** Jiping Xu, Manli Liu, Quan Liu

**Affiliations:** State Key Laboratory of Ophthalmology, Zhongshan Ophthalmic Center, Sun Yat-Sen University, Guangdong Provincial Key Laboratory of Ophthalmology and Visual Science, Guangdong Provincial Clinical Research Center for Ocular Diseases, Guangzhou, China

**Keywords:** contrast sensitivity, NeuroVision, small incision ienticule extraction, visual, visual training

## Abstract

**Objective:**

To study the improvement of subjective visual quality in patients with a small amount of residual refraction and poor visual quality after small incision ienticule extraction (SMILE) surgery through visual cortex training.

**Methods:**

Eleven subjects (22 eyes) aged 18–40 years who had undergone SMILE >6 months previously were prospectively enrolled. Subjects were trained 30 times, with the pre-training period as the baseline. Untrained patients from the same period were enrolled as controls.

**Results:**

The contrast sensitivity, best corrected visual acuity (BCVA), glare symptoms, and stereoscopic vision of the subjects was improved after training compared with before training. Compared with the eye with poor vision before training, the contrast sensitivity of the eye with good vision before training improved after training. There was no significant change in the no corrected visual acuity (NCVA) and refraction before and after training. After 12 months, contrast sensitivity was better in the visual cortex training group.

**Conclusion:**

For patients with a small amount of residual refractive error and reduced subjective visual quality after SMILE, RevitalVision training improved subjective visual function, particularly contrast sensitivity and related visual performance, without measurable changes in objective optical quality parameters.

**Clinical trial registration:**

Identifier [ChiCTR1800014796].

## 1 Introduction

Small-incision lenticule extraction (SMILE) is a frequently used surgical technique in the correction of refractive errors, which has grown in esteem among refractive surgeons and myopic patients globally in the past few years (Miao et al., 2017). The procedure is carried out using the 500 kHz VisuMax femtosecond laser (Carl Zeiss Meditec AG), whereby a stromal lenticule is generated and extracted through a 2–3 mm peripheral incision, while maintaining the anterior corneal architecture more or less intact (Chen et al., 2023). With greater utilization, there have been more reported adverse effects such as glare, vision fluctuation, and halos in the immediate months following surgery (Du et al., 2023). A study by Hjortdal et al. (2012) reported that 20% of eyes post-SMILE had ≥0.50 D and 6% had ≥1.00 D of residual refractive error at 3 months in patients with moderate-to-high myopia [mean refractive spherical equivalent (MRSE) −7.19 ± 1.30 D].

RevitalVision is a personalized, computer-based visual perceptual learning (VPL) program designed to improve visual performance through repeated stimulation of the visual cortex (Tan and Fong, 2008; Yalcin and Balci, 2014). The training protocol employs Gabor patch stimuli presented under adaptive contrast conditions together with lateral masking, a psychophysical paradigm that enhances neuronal responses by optimizing interactions among orientation-selective neurons in the primary visual cortex (Polat et al., 2004). Repetitive training induces experience-dependent cortical plasticity, thereby improving contrast sensitivity and visual discrimination without altering the optical properties of the eye. Previous studies have demonstrated that RevitalVision and similar perceptual learning approaches can improve visual acuity and contrast sensitivity in patients with amblyopia, low myopia, presbyopia, keratoconus, and other conditions associated with reduced visual function (Liza et al., 2022; Samant et al., 2026; Tang et al., 2025).

Although SMILE provides excellent refractive outcomes for most patients, a small proportion continue to experience reduced subjective visual quality despite satisfactory refractive correction and stable ocular optics (Li et al., 2025; Zhou et al., 2023). Because these symptoms are often disproportionate to objective optical findings, cortical visual adaptation may represent an important therapeutic target. Therefore, we investigated whether visual cortex training could improve subjective visual function in patients with residual refractive error after SMILE surgery.

In this study, we implemented RevitalVision visual cortex training in patients with a limited amount of residual refraction and low vision quality outcomes after the procedure of SMILE surgery. We aimed to assess the effects training had on visual acuity, residual refraction, contrast sensitivity, stereopsis, mesopic vision, glare testing, high-order aberrations, and other objective measures of visual quality pre- and post-training. We additionally enrolled a comparison group of subjects that did not complete RevitalVision training during the same timeframe for between-group comparisons in order to further assess the efficacy of the training.

We hypothesized that 30 sessions of NeuroVision visual cortex training would significantly improve contrast sensitivity, visual acuity, and subjective visual quality in patients with residual refractive error post-SMILE, with greater improvements observed in eyes with better baseline visual quality.

## 2 Methods

### 2.1 Subjects

The study protocol has been approved by the Institutional Review Board of Zhongshan Ophthalmic Center, Sun Yat-sen University (Approval Number: 2023KYPJ025). Informed consent was obtained from all subjects. Eleven patients aged 18–40 years who had undergone SMILE at the Zhongshan Eye Center, Sun Yat-sen University, for more than 6 months were enrolled.

Inclusion criteria for cortical visual training were: ① Age 18–40; gender is not limited; ② subjects underwent surgery that was conducted more than 6 months ago; ③ subjects are unhappy with their surgical outcome and have low contrast sensitivity; ④ residual SPH ≤ −1.50D, CYL ≤ −1.00D. Exclusion criteria included: ① subjects who have disabilities to their lifestyle caused by an illness, medication, or emotional state; ② subjects who have had a migraine or seizure; ③ subjects presently participating in any study that would directly or indirectly affect the outcomes of this study; ④ subjects with a previous history of mental illness; ⑤ combined with moderate/severe postoperative dry eyes, mite blepharitis, and other ocular surface diseases affecting visual quality. All subjects underwent a follow-up examination within 12 months as a long-term training outcome. For the control group, patients who were unhappy with their SMILE surgery outcome after 6 months or longer and did not receive RevitalVision training were submitted for retrospective enrollment using the same inclusion/exclusion criteria. Subsequently, age and sex-matched participants were selected from the pool of eligible patients during the same study period to minimize selection bias. Nevertheless, because the control cohort was retrospectively enrolled rather than prospectively randomized, residual selection bias and unmeasured confounding cannot be completely excluded.

### 2.2 NeuroVision training

The RevitalVision is an interactive computer that delivers individualized visual training by combining Gabor patches with lateral-masking technology. The patient sits in a darkened room 5 feet from the monitor and responds to tasks with a mouse. On each trial, the software presents two stimulus panels in random order; both contain Gabor patches, but one carries the target configuration specified for that task. The patient must identify the correct panel. A correct response lowers the target contrast for the next trial, making the task harder; an incorrect response raises the contrast, making it easier.

After every session, the program uploads the performance data via the Internet to NeuroVision servers in Singapore. A proprietary algorithm analyses the results, generates new visual-perceptual-training (VPT) parameters, and downloads them for the next session. Each session lasts ≈ 30 min; training is scheduled three times a week for a total of 30 sessions (Durrie and McMinn, 2007). The training is conducted at our eye center and is guided by a dedicated doctor.

### 2.3 Visual quality assessment

The standard logarithmic visual acuity chart was used to test no corrected visual acuity (NCVA) and best corrected visual acuity (BCVA). The CSV-1000E (VectorVision, USA) was used to record contrast sensitivity, with detection parameters including Ap, Bp, Cp, Dp, Ap + g, Bp + g, Cp + g, Dp + g, Am, Bm, Cm, Dm, Am+g, Bm + g, Cm + g, and Dm + g. Ap, Bp, Cp, Dp indicate the contrast sensitivity at low (~3 cpd), medium-low (~6 cpd), medium-high (~12 cpd), and high (~18 cpd) spatial frequencies under normal bright light conditions, while Am, Bm, Cm, Dm correspond to the results at the same frequencies but under low-light conditions. Parameters with “+g” represent tests conducted under glare conditions, assessing the impact of glare on visual quality. The OCULUS B4 (RevitalVision, China) was used to obtain the data of visual acuity, stereotest-6 m, stereotest-40 cm, contrast test, mesopic vision, and glare testing.

The phoropter was used to measure residual refraction. The Optical Quality Analysis System (OQAS) was used to obtain the following objective visual quality data: objective scattering index (OSI), Strehl Ratio (SR), modulation transfer function cutoff frequency (MTFcutoff), Predicted VA9% (VA9), Predicted VA20% (VA20), and Predicted VA100% (VA100). The high order aberration was detected using an visual quality analyzer (iTrace, Tracy, USA) with detection parameters including Z2 C3, Z2 C4, Z2 C5, Z2 C6, Z2 C7, Z2 C8, Z2 C9, Z2 C12 (Z2 C3 represents second-order aberration; Z2 C4 represents second-order astigmatism, specifically astigmatism along the horizontal axis; Z2 C5 represents second-order astigmatism, specifically astigmatism along oblique axes; Z2 C6 represents third-order aberration, specifically vertical coma; Z2 C7 represents third-order aberration, specifically horizontal coma; Z2 C8 represents third-order aberration, specifically vertical trefoil. Z2 C9 represents third-order aberration, specifically horizontal trefoil; Z2 C12 represents fourth-order aberration) (Vasudevan et al., 2015).

### 2.4 Primary and secondary outcomes

The primary outcome measures were pre-specified as: (1) change in contrast sensitivity (measured by the CSV-1000E across four spatial frequencies under photopic and mesopic conditions), and (2) change in BCVA. These were selected as primary outcomes because they represent the most direct measures of visual function that are expected to be influenced by cortical visual training.

Secondary outcome measures included: (1) stereopsis (stereotest-6 m and stereotest-40 cm); (2) glare testing; (3) visual acuity (NCVA and visual acuity score from OCULUS B4); (4) objective visual quality parameters (OSI, SR, MTFcutoff, and predicted VA); (5) residual refraction (spherical lens, cylindrical lens, and SE); and (6) higher-order aberrations (Zernike coefficients). These secondary outcomes were considered exploratory, and findings should be interpreted with appropriate caution.

### 2.5 Statistical analysis

All analyses were performed in SPSS 26.0. Continuous variables are presented as Mean ± Standard Deviation (SD). To compare the difference between the two groups, we used an independent-samples *t*-test; within each group, the baseline vs. follow-up difference was assessed with a paired *t*-test. A repeated-measures ANOVA (time × group) was also run to examine main effects and interactions; when df > 1, the Huynh–Feldt epsilon correction was applied. Given the exploratory nature of this study and the multiplicity of outcomes assessed, we applied the Benjamini-Hochberg false discovery rate (FDR) correction for multiple comparisons within each family of related outcomes (subjective visual quality metrics, objective visual quality metrics, and subgroup analyses). Adjusted *p*-values (*q*-values) are reported alongside uncorrected values. Two-tailed *p* values < 0.05 were considered statistically significant.

### 2.6 Sample size estimation

Based on the available pre-trial data from RevitalVision’s prior clinical investigations, sample size estimation was conducted prior to the initiation of this study. In a previous training cohort using the same protocol, the mean improvement in BCVA following completion of the full-course visual cortex training was −0.1 (SD 0.05), whereas the mean change in BCVA among the control population who did not receive NeuroVision training was −0.05 (SD 0.05). Sample size calculation for two independent-sample *t*-tests was performed using G*Power software (version 3.1.9), with a two-sided significance level (α) set at 0.05 and a statistical power (1 − β) of 0.80. The calculation yielded a minimum required sample size of 10 subjects per group. Nevertheless, we acknowledge that the final analysis was underpowered for subgroup analyses and multivariate modeling.

## 3 Results

### 3.1 Visual quality after NeuroVision training for all eyes

As shown in Tables 1, 2, BCVA (*p* = 0.018, *q* = 0.200), Visual acuity (*p* = 0.009, *q* = 0.200), glare testing (*p* = 0.032, *q* = 0.252), Cp + g (*p* = 0.019, *q* = 0.200), and Bm (*p* = 0.036, *q* = 0.252) during training were significantly improved compared to before training; however, these differences did not retain statistical significance after FDR correction for multiple comparisons. After 30 sessions of training, Ap (*p* = 0.045, *q* = 0.210), Dp (*p* = 0.023, *q* = 0.182), Ap + g (*p* = 0.011, *q* = 0.154), Cp + g (*p* = 0.026, *q* = 0.182), Dp + g (*p* = 0.001, *q* = 0.042), Cm (*p* = 0.011, *q* = 0.154), stereotest-6 m (*p* = 0.039, *q* = 0.205), stereotest-40 cm (*p* = 0.021, *q* = 0.182), and Contrast test (*p* = 0.032, *q* = 0.192) showed significant improvement compared to before training. After FDR correction, only Dp + g (*q* = 0.042) remained statistically significant. Compared to the “during training” results, only BCVA (*p* = 0.038, *q* = 0.317), Bp (*p* = 0.049, *q* = 0.317), Cm + g (*p* = 0.023, *q* = 0.317), and Visual acuity (*p* = 0.043, *q* = 0.317) were significantly improved after training, but none retained statistical significance after FDR correction.

**Table 1:** Objective visual quality related parameters of all eyes before, during, and after vision training.

Items	Before training	During training	After training
Spherical lens	−0.31 ± 0.38	−0.31 ± 0.53	−0.33 ± 0.61
Cylindrical lens	−0.38 ± 0.29	−0.39 ± 0.32	−0.38 ± 0.32
SE	−0.49 ± 0.37	−0.50 ± 0.53	−0.51 ± 0.60
OSI	0.98 ± 0.67	0.98 ± 0.74	0.73 ± 0.47
SR	0.22 ± 0.05	0.23 ± 0.06	0.23 ± 0.05
MTFcutoff	40.13 ± 8.94	40.93 ± 11.24	47.46 ± 6.55
VA100	1.37 ± 0.37	1.37 ± 0.37	1.58 ± 0.21
VA20	0.99 ± 0.34	0.99 ± 0.34	1.14 ± 0.33
VA9	0.61 ± 0.23	0.61 ± 0.23	0.63 ± 0.21
Z2 C3	0.08 ± 0.06	0.07 ± 0.06	0.07 ± 0.06
Z2 C4	0.57 ± 0.54	0.48 ± 0.40	0.49 ± 0.42
Z2 C5	0.13 ± 0.09	0.11 ± 0.07	0.13 ± 0.11
Z2 C6	0.02 ± 0.01	0.04 ± 0.03	0.03 ± 0.02
Z2 C7	0.04 ± 0.03	0.04 ± 0.02	0.03 ± 0.02
Z2 C8	0.03 ± 0.02	0.04 ± 0.03	0.02 ± 0.02
Z2 C9	0.02 ± 0.01	0.03 ± 0.03	0.02 ± 0.01
Z2 C12	0.02 ± 0.01	0.02 ± 0.02	0.02 ± 0.01

*q*-values represent Benjamini-Hochberg false discovery rate (FDR)-adjusted values within each family of related outcomes. An FDR threshold of *q* < 0.05 was considered statistically significant, with significant values presented in bold. Compared with before training: *q < 0.05, **q < 0.01 for during training; #*q* < 0.05, ##*q* < 0.01 for after training.

**Table 2:** Subjective visual quality related parameters of all eyes before, during and after vision training.

Items	Before training	During training	After training
NCVA	−0.01 ± 0.09	−0.03 ± 0.10	−0.02 ± 0.08
BCVA	−0.07 ± 0.08	−0.10 ± 0.08	−0.10 ± 0.08
Ap	1.46 ± 0.19	1.49 ± 0.17	1.59 ± 0.07
Bp	1.52 ± 0.28	1.59 ± 0.27	1.65 ± 0.22
Cp	1.11 ± 0.24	1.17 ± 0.32	1.25 ± 0.29
Dp	0.69 ± 0.26	0.80 ± 0.32	0.92 ± 0.23
Ap + g	1.56 ± 0.16	1.63 ± 0.19	1.65 ± 0.08
Bp + g	1.65 ± 0.23	1.66 ± 0.25	1.74 ± 0.23
Cp + g	1.13 ± 0.30	1.29 ± 0.32*	1.33 ± 0.19
Dp + g	0.75 ± 0.27	0.81 ± 0.34	1.00 ± 0.16#
Am	1.38 ± 0.20	1.48 ± 0.21	1.43 ± 0.13
Bm	1.24 ± 0.39	1.40 ± 0.32	1.37 ± 0.51
Cm	0.51 ± 0.34	0.61 ± 0.43	0.93 ± 0.29
Dm	0.36 ± 0.27	0.42 ± 0.32	0.53 ± 0.21
Am+g	1.42 ± 0.22	1.49 ± 0.20	1.48 ± 0.05
Bm + g	1.17 ± 0.46	1.16 ± 0.60	1.43 ± 0.21
Cm + g	0.51 ± 0.38	0.48 ± 0.45	0.89 ± 0.37#
Dm + g	0.29 ± 0.29	0.33 ± 0.28	0.56 ± 0.24
Visual acuity	0.84 ± 0.24	0.97 ± 0.31	0.92 ± 0.20
stereotest 6 m	200.45 ± 142.37	199.09 ± 204.04	138 ± 80.66
stereotest 40 cm	129.55 ± 158.92	108.18 ± 161.96	68.00 ± 33.60
Contrast test	22.73 ± 15.64	24.20 ± 20.78	13.75 ± 4.29
mesopic vision	18.35 ± 9.27	17.34 ± 9.80	9.84 ± 9.61
glare testing	20.05 ± 8.37	16.34 ± 10.13	13.47 ± 10.73

*q*-values represent Benjamini-Hochberg false discovery rate (FDR)-adjusted values within each family of related outcomes. An FDR threshold of *q* < 0.05 was considered statistically significant, with significant values presented in bold. Compared with before training: *q < 0.05, **q < 0.01 for during training; #*q* < 0.05, ##*q* < 0.01 for after training.

### 3.2 The impact of eye vision before training on NeuroVision training benefits

As shown in Table 3, we showed the parameters with significant improvements for good and bad eyes. The complete data are shown in Supplementary Table S1. After 30 sessions of visual training, Cp (*p* = 0.016, *q* = 0.302), Ap + g (*p* = 0.016, *q* = 0.302), Bp + g (*p* = 0.033, *q* = 0.302), Dp + g (*p* = 0.034, *q* = 0.302), Cm (*p* = 0.044, *q* = 0.308), Cm + g (*p* = 0.036, *q* = 0.302) showed significant improvement, but none retained statistical significance after FDR correction.

**Table 3:** Vision vision-related parameters of the better and worse eyes before and after vision training.

Items	Better eyes		Worse eyes	
	Before training	After training	Before training	After training
Cp	1.26 ± 0.15	1.46 ± 0.08	0.96 ± 0.22	1.05 ± 0.28
Ap + g	1.58 ± 0.17	1.66 ± 0.07	1.54 ± 0.16	1.63 ± 0.10
Bp + g	1.63 ± 0.25	1.81 ± 0.12	1.67 ± 0.21	1.66 ± 0.30
Dp + g	0.86 ± 0.26	1.1 ± 0.10	0.64 ± 0.24	0.9 ± 0.14
Cm	0.62 ± 0.35	1.15 ± 0.15	0.39 ± 0.31	0.70 ± 0.21
Cm + g	0.60 ± 0.41	1.08 ± 0.20	0.42 ± 0.34	0.70 ± 0.43

*q*-values represent Benjamini–Hochberg false discovery rate (FDR)-adjusted values within each family of related outcomes. An FDR threshold of *q* < 0.05 was considered statistically significant, with significant values presented in bold. Compared with before training: *q < 0.05, **q < 0.01 for during training; #*q* < 0.05, ##*q* < 0.01 for after training.

For the eyes with poor vision before NeuroVision training, compared with before training, after 30 sessions of training, Ap (*p* = 0.034, *q* = 0.703), Dp + g (*p* = 0.018, *q* = 0.703) were significantly improved, but none retained statistical significance after FDR correction. As for left eyes and right eyes, dominant eyes and non-dominant eyes, there was no significant difference in the visual benefits of training (Supplementary Tables S2, S3).

### 3.3 Comparison of visual quality between groups at baseline and 12-month follow-up

Visual quality at baseline and at the 12-month follow-up was compared between the two groups. At baseline, no significant differences were observed in either subjective or objective visual quality parameters between groups (Supplementary Tables S4, S5). After 12 months, the training group exhibited significantly better subjective visual quality than the control group, as indicated by a higher MTF cut-off (*p* = 0.004, *q* = 0.068) (Supplementary Table S6). In the objective assessment, the training group also outperformed the control group in the following metrics: Ap + g (*p* = 0.035, *q* = 0.093), Cp + g (*p* = 0.007, *q* = 0.054), Dp + g (*p* = 0.005, *q* = 0.054), Am (*p* = 0.049, *q* = 0.106), Cm (*p* = 0.003, *q* = 0.054), Am+g (*p* = 0.025, *q* = 0.075), Cm + g (*p* = 0.016, *q* = 0.055), Dm + g (*p* = 0.012, *q* = 0.055), the 40-cm stereo test (*p* = 0.009, *q* = 0.054), and the contrast test (*p* = 0.016, *q* = 0.055) (Supplementary Table S7). However, these differences did not retain statistical significance after FDR correction for multiple comparisons. For all findings, we have also reported effect sizes (Cohen’s d for paired comparisons) and 95% confidence intervals for the mean differences to better convey the magnitude and clinical significance of observed improvements.

## 4 Discussion

SMILE is a frequently performed refractive surgery; however, only a small number of patients experience a visual quality decrease as a result of residual refraction, glare, fluctuations in vision, and halos (Du et al., 2023). VPL refers to training that is specific to a task that enhances visual performance; it is thought to reflect plasticity in the visual system and in the broader brain networks underlying perception and behaviour (Sagi, 2011; Vasudevan et al., 2015). VPL protocols have been implemented to improve vision in clinical contexts, including amblyopia, myopia, aging, and presbyopia (DeLoss et al., 2015; Durrie and McMinn, 2007; Levi and Li, 2009; Polat et al., 2012). Previous studies have shown that NeuroVision Technology improves visual acuity and contrast sensitivity in individuals with low myopia (Lin et al., 2010; Tan and Fong, 2008), and undercorrected visual acuity and contrast sensitivity in myopic children (Ian et al., 2008), and visual function in patients with keratoconus (Tang et al., 2025).

In our study, we also found that NeuroVision training may improve subjective visual performance in patients with residual refractive error after SMILE surgery, particularly by enhancing contrast sensitivity and other perceptual measures, despite the absence of significant changes in objective optical quality.

The existing literature on visual perceptual learning has primarily focused on the improvement of binocular visual quality of patients (Camilleri et al., 2014; Leal Vega et al., 2022; Namgung et al., 2024; Tang et al., 2025), but insufficient attention has been paid to the effects of the eye differences that may influence the benefits of training. We prospectively explored the effect of eye difference on the improvement of visual quality as a result of NeuroVision treatment. However, the contrast sensitivity of the eye with better visual quality prior to NeuroVision treatment was higher than that of the eye with poorer visual quality prior to NeuroVision training, indicating that the eye with better visual quality benefited more from identical training. This suggests that the visual cortex may not give equal weight to the input of the two eyes, resulting in asymmetric function of the two eyes and visual eye preference (Chan and Chang, 2022; Kam and Chang, 2021; Ooi and He, 2020). This may also suggest that in future studies, it may be pertinent to train the eyes with the different visual qualities separately. This is similar to the classical amblyopic monocular training modality. In the treatment of the amblyopic patient, patching treatment and monocular training can significantly improve the patient’s visual function by patching the fellow eye to promote the use of the amblyopic eye (He et al., 2023; Jia et al., 2018; Kelly et al., 2016; Wallace et al., 2006). There is reason to believe that patients with different binocular visual quality can improve their visual quality more optimally by using NeuroVision treatment.

There are a number of limitations to this study. First, the small sample size was due to only around 5% of patients experiencing poor visual quality affecting their daily living after performing rehabilitative surgery (Miao et al., 2015; Pedersen et al., 2015; Shen et al., 2016; Yin et al., 2021). Second, visual training was time-consuming and labor-intensive, which resulted in fewer patients willing to undergo training. The small sample size (*n* = 11) limits the generalizability of our findings to broader populations, particularly those with varying degrees of residual refractive error, different ages, or diverse baseline visual function profiles. Therefore, our results should be considered preliminary and hypothesis-generating rather than definitive. Third, the control group was retrospectively enrolled rather than prospectively randomized, which may introduce selection bias despite matching for age and sex. Although we carefully applied identical inclusion/exclusion criteria to both groups, unmeasured confounders—such as differences in patient motivation, lifestyle factors, or baseline psychological status—cannot be excluded. Therefore, comparisons between groups should be interpreted with caution and considered supportive of the primary within-subject findings. The decision not to use a prospective parallel control arm was based on the practical consideration that patients who were dissatisfied with their surgical outcomes were highly motivated to receive training, making randomization challenging due to ethical concerns regarding withholding a potentially beneficial intervention. Nonetheless, we recognize that a randomized controlled design represents the gold standard for evidence generation, and we have acknowledged this as a limitation. Fourth, because both eyes from individual participants were included in several analyses, inter-eye correlation may have affected statistical independence. Given the limited sample size, more sophisticated approaches such as mixed-effects modeling were unlikely to provide stable estimates. Future studies with larger cohorts should incorporate subject-level or mixed-effects analyses together with randomized controlled designs.

## 5 Conclusion

For patients with residual refraction after SMILE, NeuroVision training appears to improve subjective visual quality, particularly contrast sensitivity, by enhancing neural adaptation. These findings suggest that NeuroVision treatment may represent an effective approach for improving visual performance in individuals with residual refractive errors post-SMILE, although future randomized controlled trials with larger sample sizes are warranted to confirm these observations.

## Data Availability

The original contributions presented in the study are included in the article/Supplementary material, further inquiries can be directed to the corresponding author.
