# Supplementary material for: NeuroVision training may improve subjective visual quality in patients with residual refractive error after SMILE: a prospective clinical trial
Source: Front Neurosci. 2026 Sep 9;20:1852368. doi: 10.3389/fnins.2026.1852368 (PMC13597798; doi:10.3389/fnins.2026.1852368)
Supplement: Supplementary file 1 [file Supplementary_file_1.docx]

Table S1: Vision-related parameters of the better and the worse eyes before, during, and after vision training

| Items | Better eyes | | | | Worse eyes | | |  |
| --- | --- | --- | --- | --- | --- | --- | --- | --- |
|  | Before training | During training | After training | Before training | | During training | After training | |
| NCVA | -0.05±0.08 | -0.05±0.11 | -0.06±0.09 | 0.03±0.09 | | 0.00±0.08 | 0.02±0.06 | |
| BCVA | -0.09±0.08 | -0.12±0.07 | -0.16±0.04 | -0.05±0.07 | | -0.07±0.08 | -0.03±0.04 | |
| Spherical lens | -0.25±0.42 | -0.27±0.51 | -0.40±0.6 | -0.36±0.34 | | -0.34±0.57 | -0.25±0.68 | |
| Cylindrical lens | -0.27±0.26 | -0.32±0.30 | -0.35±0.29 | -0.48±0.28 | | -0.45±0.33 | -0.40±0.38 | |
| SE | -0.39±0.38 | -0.43±0.48 | -0.58±0.49 | -0.60±0.35 | | -0.57±0.58 | -0.45±0.75 | |
| OSI | 0.89±0.44 | 0.83±0.49 | 0.63±0.44 | 1.07±0.86 | | 1.12±0.94 | 0.83±0.55 | |
| SR | 0.22±0.05 | 0.25±0.07 | 0.26±0.04 | 0.21±0.05 | | 0.21±0.07 | 0.21±0.04 | |
| MTFcutoff | 40.16±8.64 | 43.2±10.19 | 50.59±4.82 | 40.1±9.75 | | 38.66±12.37 | 44.33±7.14 | |
| VA100 | 1.44±0.34 | 1.44±0.34 | 1.68±0.15 | 1.30±0.40 | | 1.30±0.4 | 1.48±0.22 | |
| VA20 | 1.06±0.35 | 1.06±0.35 | 1.28±0.33 | 0.93±0.34 | | 0.93±0.34 | 1.00±0.32 | |
| VA9 | 0.67±0.25 | 0.67±0.25 | 0.73±0.21 | 0.54±0.21 | | 0.54±0.21 | 0.53±0.17 | |
| Z2 C3 | 0.06±0.05 | 0.05±0.02 | 0.04±0.02 | 0.10±0.07 | | 0.09±0.08 | 0.09±0.08 | |
| Z2 C4 | 0.63±0.67 | 0.44±0.43 | 0.55±0.5 | 0.51±0.39 | | 0.51±0.39 | 0.43±0.36 | |
| Z2 C5 | 0.12±0.07 | 0.11±0.07 | 0.08±0.06 | 0.14±0.11 | | 0.11±0.06 | 0.18±0.13 | |
| Z2 C6 | 0.03±0.01 | 0.04±0.03 | 0.03±0.02 | 0.02±0.01 | | 0.04±0.04 | 0.03±0.02 | |
| Z2 C7 | 0.03±0.02 | 0.03±0.02 | 0.02±0.02 | 0.04±0.03 | | 0.04±0.02 | 0.03±0.03 | |
| Z2 C8 | 0.03±0.03 | 0.03±0.03 | 0.02±0.02 | 0.02±0.02 | | 0.04±0.03 | 0.02±0.02 | |
| Z2 C9 | 0.02±0.02 | 0.02±0.01 | 0.02±0.02 | 0.02±0.01 | | 0.03±0.03 | 0.02±0.01 | |
| Z2 C12 | 0.02±0.01 | 0.02±0.01 | 0.02±0.01 | 0.02±0.01 | | 0.02±0.02 | 0.01±0.02 | |
| Ap | 1.51±0.18 | 1.48±0.19 | 1.57±0.08 | 1.41±0.20 | | 1.49±0.16 | 1.60±0.06 | |
| Bp | 1.54±0.33 | 1.63±0.28 | 1.73±0.19 | 1.51±0.22 | | 1.54±0.27 | 1.57±0.23 | |
| Cp | 1.26±0.15 | 1.24±0.31 | 1.46±0.08 | 0.96±0.22 | | 1.11±0.33 | 1.05±0.28 | |
| Dp | 0.76±0.24 | 0.75±0.36 | 1.08±0.16 | 0.63±0.27 | | 0.84±0.28 | 0.77±0.18 | |
| Ap+g | 1.58±0.17 | 1.65±0.21 | 1.66±0.07 | 1.54±0.16 | | 1.62±0.17 | 1.63±0.1 | |
| Bp+g | 1.63±0.25 | 1.69±0.23 | 1.81±0.12 | 1.67±0.21 | | 1.62±0.27 | 1.66±0.3 | |
| Cp+g | 1.27±0.24 | 1.31±0.36 | 1.48±0.08 | 0.98±0.28 | | 1.27±0.29 | 1.18±0.14 | |
| Dp+g | 0.86±0.26 | 0.84±0.41 | 1.1±0.10 | 0.64±0.24 | | 0.79±0.27 | 0.9±0.14 | |
| Am | 1.37±0.21 | 1.47±0.17 | 1.52±0.06 | 1.39±0.20 | | 1.48±0.24 | 1.34±0.11 | |
| Bm | 1.33±0.28 | 1.47±0.33 | 1.60±0.17 | 1.15±0.48 | | 1.34±0.32 | 1.14±0.64 | |
| Cm | 0.62±0.35 | 0.63±0.44 | 1.15±0.15 | 0.39±0.31 | | 0.60±0.44 | 0.70±0.21 | |
| Dm | 0.41±0.28 | 0.35±0.29 | 0.67±0.08 | 0.30±0.27 | | 0.48±0.35 | 0.38±0.21 | |
| Am+g | 1.40±0.23 | 1.47±0.20 | 1.49±0.00 | 1.44±0.22 | | 1.51±0.21 | 1.46±0.07 | |
| Bm+g | 1.15±0.60 | 1.32±0.48 | 1.55±0.11 | 1.19±0.27 | | 1.01±0.68 | 1.32±0.24 | |
| Cm+g | 0.60±0.41 | 0.63±0.47 | 1.08±0.20 | 0.42±0.34 | | 0.32±0.39 | 0.70±0.43 | |
| Dm+g | 0.32±0.28 | 0.33±0.32 | 0.60±0.21 | 0.26±0.31 | | 0.32±0.25 | 0.51±0.28 | |
| Visual acuity | 0.93±0.25 | 1.00±0.33 | 1.00±0.25 | 0.75±0.20 | | 0.94±0.3 | 0.84±0.09 | |
| stereotest 6m | 200.45±145.88 | 199.09±209.08 | 138.00±85.56 | 200.45±145.88 | | 199.09±209.08 | 138.00±85.56 | |
| stereotest 40cm | 129.55±162.84 | 108.18±165.96 | 68.00±35.64 | 129.55±162.84 | | 108.18±165.96 | 68.00±35.64 | |
| Contrast test | 23.18±20.53 | 27.27±27.74 | 12.50±3.54 | 22.27±9.58 | | 21.14±10.8 | 15.00±5.00 | |
| mesopic vision | 18.18±9.79 | 16.58±10.12 | 7.74±9.19 | 18.52±9.20 | | 18.10±9.90 | 11.94±10.60 | |
| glare testing | 20.00±8.9 | 16.25±10.59 | 14.8±11.69 | 20.09±8.24 | | 16.43±10.15 | 12.14±10.87 | |

q-values represent Benjamini-Hochberg false discovery rate (FDR)-adjusted values within each family of related outcomes. An FDR threshold of q < 0.05 was considered statistically significant, with significant values presented in bold. Compared with before training: * q < 0.05, ** q < 0.01 for during training; # q < 0.05, ## q < 0.01 for after training.

Table S2: Vision-related parameters of the dominant and non-dominant eyes before, during, and after vision training

| Items | Dominant eyes | | | | Non-dominant eyes | | |  |
| --- | --- | --- | --- | --- | --- | --- | --- | --- |
|  | Before training | During training | After training | Before training | | During training | After training | |
| NCVA | -0.03±0.09 | -0.04±0.10 | -0.01±0.07 | 0.01±0.10 | | -0.01±0.10 | -0.02±0.10 | |
| BCVA | -0.07±0.08 | -0.09±0.08 | -0.12±0.08 | -0.06±0.08 | | -0.10±0.08 | -0.07±0.07 | |
| Spherical lens | -0.32±0.34 | -0.27±0.43 | -0.30±0.76 | -0.30±0.43 | | -0.34±0.64 | -0.35±0.52 | |
| Cylindrical lens | -0.32±0.30 | -0.48±0.36 | -0.25±0.31 | -0.43±0.28 | | -0.30±0.25 | -0.50±0.31 | |
| SE | -0.48±0.33 | -0.51±0.46 | -0.43±0.75 | -0.51±0.42 | | -0.49±0.61 | -0.60±0.48 | |
| OSI | 0.87±0.47 | 0.90±0.55 | 0.63±0.44 | 1.09±0.84 | | 1.06±0.92 | 0.83±0.55 | |
| SR | 0.22±0.05 | 0.23±0.06 | 0.25±0.04 | 0.22±0.05 | | 0.22±0.08 | 0.22±0.05 | |
| MTFcutoff | 41.43±9.66 | 42.24±11.5 | 50.15±4.44 | 38.83±8.52 | | 39.62±11.5 | 44.78±7.83 | |
| VA100 | 1.42±0.38 | 1.42±0.38 | 1.65±0.13 | 1.32±0.38 | | 1.32±0.38 | 1.50±0.26 | |
| VA20 | 1.02±0.36 | 1.02±0.36 | 1.25±0.31 | 0.97±0.34 | | 0.97±0.34 | 1.03±0.36 | |
| VA9 | 0.63±0.24 | 0.63±0.24 | 0.68±0.17 | 0.58±0.24 | | 0.58±0.24 | 0.58±0.25 | |
| Z2 C3 | 0.09±0.08 | 0.08±0.08 | 0.04±0.03 | 0.07±0.05 | | 0.06±0.03 | 0.09±0.08 | |
| Z2 C4 | 0.59±0.62 | 0.41±0.34 | 0.43±0.40 | 0.56±0.47 | | 0.55±0.47 | 0.55±0.46 | |
| Z2 C5 | 0.12±0.09 | 0.12±0.07 | 0.11±0.08 | 0.13±0.10 | | 0.10±0.06 | 0.16±0.13 | |
| Z2 C6 | 0.03±0.02 | 0.04±0.03 | 0.04±0.01 | 0.02±0.01 | | 0.04±0.04 | 0.03±0.02 | |
| Z2 C7 | 0.03±0.03 | 0.04±0.03 | 0.03±0.01 | 0.04±0.02 | | 0.03±0.02 | 0.02±0.03 | |
| Z2 C8 | 0.03±0.02 | 0.03±0.02 | 0.03±0.02 | 0.03±0.02 | | 0.04±0.04 | 0.02±0.02 | |
| Z2 C9 | 0.03±0.02 | 0.02±0.02 | 0.02±0.02 | 0.02±0.01 | | 0.03±0.03 | 0.02±0.01 | |
| Z2 C12 | 0.02±0.01 | 0.02±0.01 | 0.03±0.01 | 0.02±0.01 | | 0.02±0.02 | 0.01±0.01 | |
| Ap | 1.45±0.21 | 1.49±0.16 | 1.57±0.08 | 1.47±0.19 | | 1.48±0.19 | 1.60±0.06 | |
| Bp | 1.51±0.33 | 1.66±0.25 | 1.70±0.15 | 1.54±0.22 | | 1.51±0.29 | 1.60±0.28 | |
| Cp | 1.18±0.20 | 1.24±0.23 | 1.36±0.17 | 1.04±0.27 | | 1.11±0.39 | 1.14±0.36 | |
| Dp | 0.73±0.23 | 0.78±0.31 | 0.95±0.22 | 0.65±0.30 | | 0.82±0.34 | 0.89±0.26 | |
| Ap+g | 1.54±0.15 | 1.63±0.13 | 1.66±0.07 | 1.58±0.18 | | 1.63±0.24 | 1.63±0.10 | |
| Bp+g | 1.63±0.25 | 1.69±0.24 | 1.81±0.12 | 1.67±0.21 | | 1.62±0.26 | 1.66±0.30 | |
| Cp+g | 1.23±0.25 | 1.31±0.27 | 1.46±0.08 | 1.03±0.31 | | 1.26±0.37 | 1.21±0.20 | |
| Dp+g | 0.77±0.24 | 0.82±0.37 | 1.04±0.08 | 0.72±0.30 | | 0.80±0.33 | 0.95±0.22 | |
| Am | 1.36±0.21 | 1.46±0.18 | 1.49±0.10 | 1.40±0.20 | | 1.50±0.24 | 1.37±0.13 | |
| Bm | 1.29±0.31 | 1.47±0.28 | 1.51±0.14 | 1.19±0.48 | | 1.34±0.37 | 1.23±0.71 | |
| Cm | 0.51±0.36 | 0.63±0.44 | 1.02±0.27 | 0.50±0.34 | | 0.60±0.44 | 0.83±0.31 | |
| Dm | 0.38±0.31 | 0.32±0.29 | 0.55±0.21 | 0.33±0.24 | | 0.51±0.33 | 0.51±0.24 | |
| Am+g | 1.39±0.19 | 1.47±0.17 | 1.49±0.00 | 1.45±0.26 | | 1.51±0.23 | 1.46±0.07 | |
| Bm+g | 1.10±0.61 | 1.22±0.61 | 1.52±0.08 | 1.24±0.25 | | 1.10±0.61 | 1.35±0.28 | |
| Cm+g | 0.51±0.43 | 0.49±0.44 | 0.98±0.09 | 0.50±0.34 | | 0.46±0.48 | 0.80±0.53 | |
| Dm+g | 0.20±0.29 | 0.29±0.29 | 0.5±0.08 | 0.38±0.29 | | 0.36±0.28 | 0.61±0.35 | |
| Visual acuity | 0.83±0.21 | 0.98±0.29 | 0.95±0.21 | 0.84±0.28 | | 0.95±0.34 | 0.89±0.20 | |
| Stereotest 6m | 200.45±145.88 | 199.09±209.08 | 138.00±85.56 | 200.45±145.88 | | 199.09±209.08 | 138.00±85.56 | |
| Stereotest 40cm | 129.55±162.84 | 108.18±165.96 | 68.00±35.64 | 129.55±162.84 | | 108.18±165.96 | 68.00±35.64 | |
| Contrast test | 24.55±19.81 | 23.64±20.84 | 14.00±2.24 | 20.91±10.68 | | 24.77±21.72 | 13.50±6.02 | |
| mesopic vision | 18.45±9.32 | 18.52±9.20 | 7.88±9.08 | 18.25±9.67 | | 16.16±10.68 | 11.80±10.76 | |
| glare testing | 20.27±8.32 | 18.25±9.67 | 14.94±11.5 | 19.82±8.82 | | 14.43±10.67 | 12.00±11.02 | |

q-values represent Benjamini-Hochberg false discovery rate (FDR)-adjusted values within each family of related outcomes. An FDR threshold of q < 0.05 was considered statistically significant, with significant values presented in bold. Compared with before training: * q < 0.05, ** q < 0.01 for during training; # q < 0.05, ## q < 0.01 for after training.

Table S3: Vision-related parameters of the right and left eyes before, during and after vision training

| Items | Right eyes | | | | Left eyes | | |  |
| --- | --- | --- | --- | --- | --- | --- | --- | --- |
|  | Before training | During training | After training | Before training | | During training | After training | |
| NCVA | 0.00±0.10 | -0.02±0.10 | 0.00±0.07 | -0.02±0.09 | | -0.03±0.10 | -0.03±0.10 | |
| BCVA | -0.06±0.08 | -0.08±0.09 | -0.09±0.09 | -0.07±0.08 | | -0.11±0.07 | -0.10±0.08 | |
| Spherical lens | -0.30±0.33 | -0.32±0.48 | -0.25±0.68 | -0.32±0.43 | | -0.30±0.60 | -0.40±0.60 | |
| Cylindrical lens | -0.43±0.25 | -0.48±0.36 | -0.35±0.38 | -0.32±0.32 | | -0.30±0.25 | -0.40±0.29 | |
| SE | -0.51±0.34 | -0.56±0.48 | -0.43±0.74 | -0.48±0.41 | | -0.44±0.59 | -0.60±0.50 | |
| OSI | 0.88±0.46 | 0.94±0.55 | 0.73±0.56 | 1.08±0.85 | | 1.01±0.92 | 0.73±0.46 | |
| SR | 0.22±0.06 | 0.21±0.06 | 0.25±0.04 | 0.21±0.04 | | 0.24±0.08 | 0.22±0.05 | |
| MTFcutoff | 41.55±10.2 | 39.97±11.08 | 50.23±4.36 | 38.7±7.82 | | 41.89±11.98 | 44.69±7.80 | |
| VA100 | 1.34±0.36 | 1.34±0.36 | 1.65±0.13 | 1.40±0.40 | | 1.40±0.40 | 1.50±0.26 | |
| VA20 | 0.94±0.34 | 0.94±0.34 | 1.25±0.31 | 1.04±0.36 | | 1.04±0.36 | 1.03±0.36 | |
| VA9 | 0.56±0.22 | 0.56±0.22 | 0.68±0.17 | 0.66±0.25 | | 0.66±0.25 | 0.58±0.25 | |
| Z2 C3 | 0.09±0.07 | 0.08±0.08 | 0.04±0.02 | 0.06±0.06 | | 0.06±0.03 | 0.09±0.08 | |
| Z2 C4 | 0.56±0.58 | 0.48±0.35 | 0.40±0.39 | 0.59±0.51 | | 0.48±0.47 | 0.58±0.46 | |
| Z2 C5 | 0.14±0.10 | 0.12±0.07 | 0.11±0.07 | 0.12±0.09 | | 0.09±0.06 | 0.15±0.14 | |
| Z2 C6 | 0.02±0.02 | 0.04±0.03 | 0.03±0.02 | 0.02±0.01 | | 0.05±0.04 | 0.03±0.02 | |
| Z2 C7 | 0.03±0.03 | 0.04±0.03 | 0.02±0.02 | 0.04±0.02 | | 0.03±0.02 | 0.03±0.02 | |
| Z2 C8 | 0.03±0.02 | 0.03±0.02 | 0.02±0.02 | 0.03±0.02 | | 0.04±0.04 | 0.02±0.02 | |
| Z2 C9 | 0.02±0.02 | 0.02±0.02 | 0.02±0.01 | 0.02±0.01 | | 0.03±0.03 | 0.02±0.02 | |
| Z2 C12 | 0.02±0.01 | 0.02±0.01 | 0.02±0.01 | 0.02±0.02 | | 0.02±0.02 | 0.02±0.01 | |
| Ap | 1.51±0.16 | 1.51±0.16 | 1.60±0.06 | 1.41±0.21 | | 1.46±0.19 | 1.57±0.08 | |
| Bp | 1.49±0.31 | 1.62±0.23 | 1.60±0.26 | 1.56±0.26 | | 1.55±0.32 | 1.7±0.18 | |
| Cp | 1.10±0.23 | 1.16±0.29 | 1.11±0.32 | 1.12±0.26 | | 1.18±0.36 | 1.39±0.19 | |
| Dp | 0.73±0.26 | 0.86±0.21 | 0.83±0.18 | 0.66±0.28 | | 0.74±0.40 | 1.01±0.25 | |
| Ap+g | 1.59±0.11 | 1.66±0.13 | 1.63±0.10 | 1.52±0.20 | | 1.61±0.24 | 1.66±0.07 | |
| Bp+g | 1.73±0.16 | 1.72±0.25 | 1.69±0.31 | 1.57±0.27 | | 1.59±0.24 | 1.79±0.13 | |
| Cp+g | 1.15±0.25 | 1.31±0.29 | 1.30±0.21 | 1.10±0.34 | | 1.27±0.36 | 1.36±0.20 | |
| Dp+g | 0.73±0.25 | 0.84±0.29 | 0.99±0.06 | 0.77±0.29 | | 0.78±0.40 | 1.01±0.23 | |
| Am | 1.40±0.17 | 1.53±0.21 | 1.37±0.13 | 1.36±0.23 | | 1.43±0.20 | 1.49±0.10 | |
| Bm | 1.29±0.31 | 1.41±0.32 | 1.20±0.69 | 1.19±0.48 | | 1.40±0.35 | 1.54±0.19 | |
| Cm | 0.43±0.37 | 0.52±0.46 | 0.93±0.30 | 0.58±0.32 | | 0.71±0.40 | 0.93±0.32 | |
| Dm | 0.32±0.32 | 0.42±0.35 | 0.51±0.20 | 0.39±0.23 | | 0.41±0.31 | 0.55±0.24 | |
| Am+g | 1.47±0.19 | 1.51±0.21 | 1.46±0.07 | 1.37±0.25 | | 1.47±0.20 | 1.49±0.00 | |
| Bm+g | 1.18±0.29 | 1.15±0.60 | 1.39±0.28 | 1.16±0.60 | | 1.18±0.62 | 1.48±0.14 | |
| Cm+g | 0.43±0.44 | 0.42±0.44 | 0.83±0.47 | 0.58±0.32 | | 0.53±0.48 | 0.95±0.28 | |
| Dm+g | 0.29±0.34 | 0.28±0.27 | 0.60±0.21 | 0.29±0.25 | | 0.38±0.30 | 0.51±0.28 | |
| Visual acuity | 0.81±0.21 | 0.94±0.30 | 0.97±0.19 | 0.87±0.28 | | 1.00±0.33 | 0.87±0.22 | |
| stereotest 6m | 200.45±145.88 | 199.09±209.08 | 138±85.56 | 200.45±145.88 | | 199.09±209.08 | 138.00±85.56 | |
| stereotest 40cm | 129.55±162.84 | 108.18±165.96 | 68.00±35.64 | 129.55±162.84 | | 108.18±165.96 | 68.00±35.64 | |
| Contrast test | 19.55±7.57 | 20±11.35 | 12.00±2.74 | 25.91±20.83 | | 28.41±27.19 | 15.50±5.12 | |
| mesopic vision | 18.25±9.67 | 18.31±9.56 | 8.34±8.81 | 18.45±9.32 | | 16.37±10.41 | 11.34±11.16 | |
| glare testing | 20.27±8.32 | 18.15±9.62 | 15.54±10.71 | 19.82±8.82 | | 14.52±10.75 | 11.40±11.57 | |

q-values represent Benjamini-Hochberg false discovery rate (FDR)-adjusted values within each family of related outcomes. An FDR threshold of q < 0.05 was considered statistically significant, with significant values presented in bold. Compared with before training: * q < 0.05, ** q < 0.01 for during training; # q < 0.05, ## q < 0.01 for after training.

Table S4: Objective visual quality parameters between groups at baseline

| Items | NeuroVision training group | Control group | t | p value | q-value | Cohen's d(95%CI) |
| --- | --- | --- | --- | --- | --- | --- |
| Spherical lens | -0.31±0.38 | -0.31±0.37 | 0.029 | 0.977 | 1.000 | 0.00(-0.59,0.59) |
| Cylindrical lens | -0.38±0.29 | -0.37±0.30 | -0.094 | 0.926 | 1.000 | -0.03(-0.62,0.56) |
| SE | -0.49±0.37 | -0.48±0.37 | -0.058 | 0.954 | 1.000 | -0.03(-0.62,0.56) |
| OSI | 0.98±0.67 | 0.98±0.65 | 0.013 | 0.990 | 1.000 | 0.00(-0.59,0.59) |
| SR | 0.22±0.05 | 0.22±0.05 | 0.089 | 0.93 | 1.000 | 0.00(-0.59,0.59) |
| MTFcutoff | 40.13±8.94 | 37.97±8.54 | 0.579 | 0.569 | 1.000 | 0.25(-0.35,0.84) |
| VA100 | 1.37±0.37 | 1.50±0.36 | -0.818 | 0.423 | 1.000 | -0.36(-0.95,0.24) |
| VA20 | 0.99±0.34 | 0.96±0.34 | 0.225 | 0.825 | 1.000 | 0.09(-0.50,0.68) |
| VA9 | 0.61±0.23 | 0.64±0.24 | -0.314 | 0.757 | 1.000 | -0.13(-0.72,0.46) |
| Z2 C3 | 0.08±0.06 | 0.07±0.06 | 0.257 | 0.800 | 1.000 | 0.17(-0.43,0.76) |
| Z2 C4 | 0.57±0.54 | 0.58±0.51 | -0.024 | 0.981 | 1.000 | -0.02(-0.61,0.57) |
| Z2 C5 | 0.13±0.09 | 0.12±0.09 | 0.24 | 0.813 | 1.000 | 0.11(-0.48,0.70) |
| Z2 C6 | 0.02±0.01 | 0.02±0.01 | -0.43 | 0.672 | 1.000 | 0.00(-0.59,0.59) |
| Z2 C7 | 0.04±0.03 | 0.04±0.03 | 0.301 | 0.767 | 1.000 | 0.00(-0.59,0.59) |
| Z2 C8 | 0.03±0.02 | 0.03±0.02 | 0.117 | 0.908 | 1.000 | 0.00(-0.59,0.59) |
| Z2 C9 | 0.02±0.01 | 0.02±0.01 | -1.166 | 0.258 | 1.000 | 0.00(-0.59,0.59) |
| Z2 C12 | 0.02±0.01 | 0.02±0.01 | 0.00 | 1.000 | 1.000 | 0.00(-0.59,0.59) |

An FDR threshold of q < 0.05 was considered statistically significant, with significant values presented in bold.

Table S5: Subjective visual quality parameters between groups at baseline

| Items | NeuroVision training group | Control group | t | p value | q-value | Cohen's d(95%CI) |
| --- | --- | --- | --- | --- | --- | --- |
| NCVA | -0.01±0.09 | -0.01±0.10 | 0.066 | 0.948 | 0.977 | 0.00(-0.59,0.59) |
| BCVA | -0.07±0.08 | -0.07±0.07 | -0.029 | 0.977 | 0.977 | 0.00(-0.59,0.59) |
| Ap | 1.46±0.19 | 1.47±0.17 | -0.179 | 0.86 | 0.977 | -0.06(-0.65,0.54) |
| Bp | 1.52±0.28 | 1.56±0.29 | -0.334 | 0.742 | 0.977 | -0.14(-0.73,0.45) |
| Cp | 1.11±0.24 | 1.05±0.24 | 0.558 | 0.583 | 0.977 | 0.25(-0.34,0.84) |
| Dp | 0.69±0.26 | 0.66±0.27 | 0.292 | 0.774 | 0.977 | 0.11(-0.48,0.70) |
| Ap+g | 1.56±0.16 | 1.57±0.17 | -0.103 | 0.919 | 0.977 | -0.06(-0.65,0.53) |
| Bp+g | 1.65±0.23 | 1.80±0.23 | -1.534 | 0.141 | 0.977 | -0.65(-1.26,-0.05) |
| Cp+g | 1.13±0.30 | 1.15±0.28 | -0.155 | 0.878 | 0.977 | -0.07(-0.66,0.52) |
| Dp+g | 0.75±0.27 | 0.69±0.28 | 0.499 | 0.624 | 0.977 | 0.22(-0.37,0.81) |
| Am | 1.38±0.20 | 1.25±0.21 | 1.513 | 0.146 | 0.977 | 0.63(0.03,1.24) |
| Bm | 1.24±0.39 | 1.33±0.41 | -0.504 | 0.620 | 0.977 | -0.22(-0.82,0.37) |
| Cm | 0.51±0.34 | 0.54±0.31 | -0.197 | 0.846 | 0.977 | -0.09(-0.68,0.50) |
| Dm | 0.36±0.27 | 0.38±0.30 | -0.203 | 0.841 | 0.977 | -0.07(-0.66,0.52) |
| Am+g | 1.42±0.22 | 1.35±0.24 | 0.718 | 0.481 | 0.977 | 0.30(-0.29,0.90) |
| Bm+g | 1.17±0.46 | 1.26±0.49 | -0.431 | 0.671 | 0.977 | -0.19(-0.78,0.40) |
| Cm+g | 0.51±0.38 | 0.47±0.39 | 0.262 | 0.796 | 0.977 | 0.10(-0.49,0.70) |
| Dm+g | 0.29±0.29 | 0.28±0.27 | 0.084 | 0.934 | 0.977 | 0.04(-0.56,0.63) |
| Visual acuity | 0.84±0.24 | 0.90±0.25 | -0.603 | 0.553 | 0.977 | -0.24(-0.84,0.35) |
| stereotest 6m | 200.45±82.37 | 216.52±84.60 | -0.452 | 0.656 | 0.977 | -0.19(-0.78,0.40) |
| stereotest 40cm | 129.55±68.92 | 132.91±68.17 | -0.115 | 0.910 | 0.977 | -0.05(-0.64,0.54) |
| Contrast test | 22.73±10.64 | 20.54±11.65 | 0.461 | 0.650 | 0.977 | 0.20(-0.40,0.79) |
| mesopic vision | 18.35±9.27 | 18.21±9.91 | 0.033 | 0.974 | 0.977 | 0.01(-0.58,0.61) |
| glare testing | 20.05±8.37 | 18.72±7.77 | 0.386 | 0.704 | 0.977 | 0.16(-0.43,0.76) |

An FDR threshold of q < 0.05 was considered statistically significant, with significant values presented in bold.

Table S6: Objective visual quality parameters between groups at 12-month follow-up

| Items | NeuroVision training group | Control group | t | p value | q-value | Cohen's d(95%CI) |
| --- | --- | --- | --- | --- | --- | --- |
| Spherical lens | -0.33±0.61 | -0.31±0.34 | -0.099 | 0.922 | 0.971 | -0.04(-0.63,0.55) |
| Cylindrical lens | -0.38±0.32 | -0.35±0.32 | -0.239 | 0.813 | 0.971 | -0.09(-0.69,0.50) |
| SE | -0.51±0.60 | -0.47±0.35 | -0.209 | 0.837 | 0.971 | -0.08(-0.67,0.51) |
| OSI | 0.73±0.47 | 0.93±0.68 | -0.809 | 0.429 | 0.971 | -0.34(-0.94,0.25) |
| SR | 0.23±0.05 | 0.22±0.04 | 0.282 | 0.781 | 0.971 | 0.22(-0.37,0.81) |
| MTFcutoff | 47.46±6.55 | 36.97±8.22 | 3.311 | 0.004 | 0.068 | 1.41(0.75,2.07) |
| VA100 | 1.58±0.21 | 1.40±0.38 | 1.409 | 0.179 | 0.761 | 0.59(-0.02,1.19) |
| VA20 | 1.14±0.33 | 1.00±0.33 | 0.999 | 0.330 | 0.935 | 0.42(-0.17,1.02) |
| VA9 | 0.63±0.21 | 0.62±0.23 | 0.087 | 0.932 | 0.971 | 0.05(-0.55,0.64) |
| Z2 C3 | 0.07±0.06 | 0.07±0.06 | -0.037 | 0.971 | 0.971 | 0.00(-0.59,0.59) |
| Z2 C4 | 0.49±0.42 | 0.53±0.55 | -0.208 | 0.837 | 0.971 | -0.08(-0.67,0.51) |
| Z2 C5 | 0.13±0.11 | 0.12±0.09 | 0.315 | 0.756 | 0.971 | 0.10(-0.49,0.69) |
| Z2 C6 | 0.03±0.02 | 0.02±0.01 | 1.747 | 0.107 | 0.606 | 0.63(0.03,1.24) |
| Z2 C7 | 0.03±0.02 | 0.04±0.03 | -1.096 | 0.287 | 0.935 | -0.39(-0.99,0.20) |
| Z2 C8 | 0.02±0.02 | 0.03±0.02 | -1.719 | 0.101 | 0.606 | -0.50(-1.10,0.10) |
| Z2 C9 | 0.02±0.01 | 0.02±0.01 | -0.232 | 0.819 | 0.971 | 0.00(-0.59,0.59) |
| Z2 C12 | 0.02±0.01 | 0.02±0.01 | 0.711 | 0.486 | 0.971 | 0.00(-0.59,0.59) |

An FDR threshold of q < 0.05 was considered statistically significant, with significant values presented in bold.

Table S7: Subjective visual quality parameters between groups at 12-month follow-up

| Items | NeuroVision training group | Control group | t | p value | q-value | Cohen's d(95%CI) |
| --- | --- | --- | --- | --- | --- | --- |
| NCVA | -0.02±0.08 | -0.01±0.10 | -0.283 | 0.780 | 0.783 | -0.11(-0.70,0.48) |
| BCVA | -0.10±0.08 | -0.07±0.06 | -1.026 | 0.318 | 0.402 | -0.42(-1.02,0.17) |
| Ap | 1.59±0.07 | 1.49±0.16 | 1.928 | 0.075 | 0.129 | 0.81(0.20,1.42) |
| Bp | 1.65±0.22 | 1.60±0.27 | 0.464 | 0.648 | 0.707 | 0.20(-0.39,0.80) |
| Cp | 1.25±0.29 | 1.13±0.23 | 1.103 | 0.284 | 0.388 | 0.46(-0.14,1.06) |
| Dp | 0.92±0.23 | 0.69±0.29 | 2.09 | 0.050 | 0.106 | 0.88(0.26,1.50) |
| Ap+g | 1.65±0.08 | 1.51±0.18 | 2.333 | 0.035 | 0.093 | 1.01(0.38,1.63) |
| Bp+g | 1.74±0.23 | 1.69±0.24 | 0.499 | 0.623 | 0.707 | 0.21(-0.38,0.81) |
| Cp+g | 1.33±0.19 | 1.03±0.27 | 3.016 | 0.007 | 0.054 | 1.29(0.64,1.93) |
| Dp+g | 1.00±0.16 | 0.67±0.29 | 3.243 | 0.005 | 0.054 | 1.41(0.75,2.07) |
| Am | 1.43±0.13 | 1.28±0.19 | 2.109 | 0.049 | 0.106 | 0.92(0.30,1.54) |
| Bm | 1.37±0.51 | 1.26±0.37 | 0.587 | 0.564 | 0.677 | 0.25(-0.35,0.84) |
| Cm | 0.93±0.29 | 0.51±0.29 | 3.431 | 0.003 | 0.054 | 1.45(0.78,2.11) |
| Dm | 0.53±0.21 | 0.42±0.27 | 1.086 | 0.291 | 0.388 | 0.45(-0.14,1.05) |
| Am+g | 1.48±0.05 | 1.29±0.24 | 2.592 | 0.025 | 0.075 | 1.10(0.46,1.73) |
| Bm+g | 1.43±0.21 | 1.23±0.50 | 1.211 | 0.247 | 0.371 | 0.52(-0.08,1.12) |
| Cm+g | 0.89±0.37 | 0.47±0.38 | 2.619 | 0.016 | 0.055 | 1.12(0.48,1.76) |
| Dm+g | 0.56±0.24 | 0.27±0.25 | 2.754 | 0.012 | 0.055 | 1.18(0.54,1.82) |
| Visual acuity | 0.92±0.20 | 0.95±0.23 | -0.279 | 0.783 | 0.783 | -0.14(-0.73,0.45) |
| Stereotest 6m | 138.00±80.66 | 206.70±75.62 | -2.061 | 0.053 | 0.106 | -0.88(-1.50,-0.26) |
| Stereotest 40cm | 68.00±33.60 | 132.69±63.98 | -2.969 | 0.009 | 0.054 | -1.27(-1.91,-0.62) |
| Contrast test | 13.75±4.29 | 21.89±8.96 | -2.719 | 0.016 | 0.055 | -1.16(-1.80,-0.52) |
| mesopic vision | 9.84±9.61 | 17.89±9.60 | -1.966 | 0.063 | 0.116 | -0.84(-1.45,-0.22) |
| glare testing | 13.47±10.73 | 20.51±8.20 | -1.729 | 0.100 | 0.16 | -0.74(-1.35,-0.13) |

An FDR threshold of q < 0.05 was considered statistically significant, with significant values presented in bold.
